# Mild-stretch mechanical ventilation upregulates toll-like receptor 2 and sensitizes the lung to bacterial lipopeptide

**DOI:** 10.1186/cc10330

**Published:** 2011-07-27

**Authors:** Pierre-Emmanuel Charles, Pierre Tissières, Saber Davide Barbar, Delphine Croisier, Julien Dufour, Irène Dunn-Siegrist, Pascal Chavanet, Jérôme Pugin

**Affiliations:** 1 Laboratoire Interactions Muqueuses Agents pathogènes (L.I.M.A.), EA562, U.F.R. Médecine, Université de Bourgogne, 7 Bd Jeanne d'Arc, 21000 Dijon, France; 2 Intensive Care Laboratory, University Hospitals of Geneva, Rue Gabrielle-Perret-Gentil 4, 1211 Geneva 14, Switzerland; 3 Department of Microbiology and Molecular Medicine, Faculty of Medicine, University Hospitals of Geneva, Rue Gabrielle-Perret-Gentil 4, 1211 Geneva 14, Switzerland

## Abstract

**Introduction:**

Mechanical ventilation (MV) could prime the lung toward an inflammatory response if exposed to another insult such as bacterial invasion. The underlying mechanisms are not so far clear. Toll-like receptors (TLRs) allow the host to recognize selectively bacterial pathogens and in turn to trigger an immune response. We therefore hypothesized that MV modulates *TLR2 *expression and in turn modifies responsiveness to agonists such as bacterial lipopeptide (BLP).

**Method:**

Both *in vitro *and *in vivo *experiments were conducted. First, *TLR2 *expression and protein were measured in the A549 pulmonary epithelial cell line submitted to 8-hour cyclic stretch (20% elongation; 20/minute rate). After a 24-hour period of cyclic stretch, the inflammatory response of the A549 cells to the synthetic BLP, Pam_3_CSK_4_, was tested after 8 hours of exposure. In a second set of experiments, healthy anesthetized and paralyzed rabbits were submitted to 8-hour MV (tidal volume = 12 ml/kg, zero end-expiratory pressure; FIO_2 _= 50%; respiratory rate = 20/minute) before being sacrificed for *TLR2 *lung expression assessment. The lung inflammatory response to BLP was then tested in animals submitted to 24-hour MV before being sacrificed 8 hours after the tracheal instillation of Pam_3_CSK_4_.

**Results:**

Cyclic stretch of human pulmonary epithelial cell lines increased both *TLR2 *mRNA and protein expression. Cells submitted to cyclic stretch also increased IL-6 and IL-8 secretion in response to Pam_3_CSK_4_, a classical TLR2 ligand. A mild-stretch MV protocol induced a 60-fold increase of *TLR2 *mRNA expression in lung tissue when compared with spontaneously breathing controls. Moreover, the combination of MV and airway exposure to Pam_3_CSK_4 _acted synergistically in causing lung inflammation and injury.

**Conclusions:**

Mild-stretch MV increases lung expression of *TLR2 *and sensitizes the lung to bacterial TLR2 ligands. This may account for the propensity of mechanically ventilated patients to develop acute lung injury in the context of airway bacterial colonization/infection.

## Introduction

Bacterial superinfection of the lung is a frequent complication in critically ill patients requiring mechanical ventilation (MV) [1]. An incidence of ventilator-associated pneumonia of up to 30% has been reported in patients requiring prolonged MV. Among the bacteria causing ventilator-associated pneumonia, *Staphylococcus aureus *is a leading source of infection and mortality [2]. Both *in vitro *and *in vivo *experimental studies have demonstrated that MV could activate lung cells and induce a proinflammatory response. Although low-stretch MV does not lead to apparent tissue damage or inflammation, it could prime airway cells to respond massively to a second proinflammatory insult such as lipopolysaccharide (LPS) [3-6]. The subsequent release of large amounts of cytokines is probably responsible for substantial lung injury, particularly through the recruitment of neutrophils mediated by IL-8 secretion [7].

Little is known, however, about the role of Gram-positive bacterial products in this context. In several animal studies, MV has been shown to impair lung bacterial clearance, to increase lung injury and to promote pulmonary-to-systemic microbial translocation [8-11]. However, the link between these different observations remains to be established.

A prompt immune lung response is necessary to ensure microbial clearance. Early pathogen recognition via the so-called pathogen recognition receptors is considered a first and key step in this process. Among the pathogen recognition receptors, toll-like receptors (TLRs) play an indisputable and major role in the recognition of pathogen-associated microbial patterns. The ligation of TLRs by pathogen-associated molecular patterns induces the signaling of the subsequent inflammatory response. Bacterial lipopeptides (BLPs) are cell wall components of both Gram-positive and Gram-negative bacteria and have been recognized to activate TLR2 in a heterodimeric association with TLR1 or TLR6 [12,13].

TLR2 is mainly expressed by myeloid cells, but epithelial cells from various tissues can also express the receptor [14]. In the airway, both bronchial and alveolar epithelial cells participate in the lung immune response through their ability to produce inflammatory mediators and chemokines [15]. Whereas TLR4 is essential for cell responses to Gram-negative LPS, TLR2 is implicated in innate immune responses to Gram-positive bacteria [12,13]. For example, *Tlr2*^-/- ^mice are highly susceptible to infections caused by Gram-positive bacteria such as *S. aureus *[16]. *TLR2 *expression also seems sensitive to mechanical stress, at least in the endothelium, where it has been shown to be upregulated by various types of mechanical strains [17]. Whereas the concept of lung priming was relatively well described for LPS models depending on the TLR4 pathway [3-6,18], little is known on the possible implication of other TLRs and related bacterial ligands in MV-dependent lung injury.

To test the hypothesis that MV modulates *TLR2 *expression, we performed a set of *in vitro *and *in vivo *experiments to determine whether cyclic stretch of lung cells and MV modified the expression of *TLR2 *and to unravel the effect of MV in lung inflammatory response and injury after exposure to a classical TLR2 bacterial ligand.

## Materials and methods

### Cells and reagents

The human epithelial type II-like A549 cell line was purchased from the American Type Culture Collection (Rockville, MD, USA) and cultured in a 50/50 mixture of DMEM and F-12 nutrient mixture supplemented with 10% FCS, 2 mM L-glutamine, 10 mM HEPES, 50 U/ml penicillin, and 2 μg/ml gentamicin. The NF-κB inhibitor Sn50 and the p38 mitogen-activated protein kinase (MAPK) specific inhibitor SB203580 were purchased from Sigma (St Louis, MO, USA). The TLR2-specific agonist Pam_3_CSK_4 _lipopeptide was purchased from InvivoGen (San Diego, CA, USA).

### Cell culture and cyclic stretch

A549 epithelial cell lines were seeded onto Bioflex^® ^plates (Dunn Labortechnik, Asbach, Germany) at a density of 2 × 10^5 ^cells/well, and were cultured for 48 hours until 70 to 80% confluency was achieved. Mechanical stretch was then performed for different periods of time in fresh medium using the cell stretching apparatus FX-3000 Flexercell strain unit (Flexcell International, Hillsborough, N.C., U.S.A.) in a 37°C, 5% carbon dioxide incubator. The stretching rate was 20 cycles/minute with a square signal, a 1:1 stretch:relaxation ratio, and a 20% maximal equibiaxial elongation, as previously described [7]. In some experiments, Pam_3_CSK_4 _was added to the medium (final concentration 1 μg/ml) for an additional 8 hours. At the end of the experiments, conditioned supernatants were immediately collected and kept frozen at -80°C. IL-6 and IL-8 were quantified by ELISA (Endogen, Woburn, MA, USA) in thawed conditioned supernatants. Limits of detection were 45 pg/ml for IL-6 and 65 pg/ml for IL-8.

### Quantitative RT-PCR

After stretching, A549 cells were harvested and kept in TriZol (Invitrogen, San Diego, CA, USA) at -70°C until the day of RNA extraction. Complementary DNA was obtained by reverse transcription using random primers, RNasin treatment, and ImProm II RT (Promega, Madison, WI, USA). Quantitative PCR was performed using the IQ5 thermocycler (Biorad, Hercules, CA, USA), and the following TaqMan probes with the universal PCR Mastermix (Applied Biosystems, Foster City, CA, USA): *MD-2*, Hs00209771_m1; *TLR4*, Hs00152939_m1; *TLR2*, Hs00610101_m1; *CD14*, Hs00169122_g1; *IL-8*, Hs00174103_m1; *IL-6*, Hs 00174131_m1; *HBD1*, Hs00608345_m1; *HBD2*, Hs00175474_m1; *HBD3*, Hs00218678_m1; 18S, Hs99999901_s1.

In rabbits, after sacrifice and exsanguination, the rib cage was opened and lung pieces were cut and RNA was then extracted using the RNA GenElute kit (Sigma). Quantitative PCR was performed using IQ Sybrgreen Supermix (Biorad). Melting curves were performed to ensure the presence of a single amplicon. The following primers were used: *rTlr2*, forward 5'-TGT CTG TCA CCG AAC CGA ATC CAC-3' and reverse 5'-TCA GGT TTT TCA GCG TCA GCA GGG-3'; *rTlr4*, forward 5'-GAG CAC CTG GAC CTT TCA AAT AAC-3' and reverse 5'-GAA CTT CTA AAC CAC TCA GCC CTT-3'; *rGapdh*, forward 5'-ATG TTT GTG ATG GGC GTG AAC C-3' and reverse 5'-CCC AGC ATC GAA GGT AGA GGA-3'; *rCd14*, forward 5'-TCT CTG TCC CCA CAA GTT CC-3' and reverse 5'-CAC CTG CTG CAG TCC AGT AA-3'; *rMd-2*, forward 5'-GGG AAC CCA AGG TTT ATT GC-3' and reverse 5'-CGT ATG CCC TTG AAG GAA AA-3'; and *rIl-8*, forward 5'-AAC CTT CCT GCT GCT TCT GA-3' and reverse 5'-TCT GCA CCC ACT TTT TCC TTG-3'.

The results are expressed as the fold induction using the ΔCt method since the static condition (*in vitro*) or the spontaneously breathing (SB) animals (*in vivo*) were always considered the baseline condition (that is, the '1' reference value)

### Flow cytometry

A549 cells stretched or kept in static conditions for 24 hours were removed from culture plates using pH 7.4 PBS containing 0.1% ethylenediamine tetraacetic acid and were used immediately for fluorescence-activated cell sorting (FACS). For each analysis, 10^6 ^cells were washed three times with 1 ml PBS containing 0.4% BSA (Sigma) at 4°C. Cells were incubated in PBS/BSA containing 5 μg/ml mouse anti-huTLR2 mAb (T2.5 clone) antibody or an isotype control antibody (mouse IgG1; Biosource, Camarillo, CA, USA), at 4°C for 30 minutes. Cells were then washed three times with PBS and resuspended PBS containing 10 μg/ml goat anti-mouse IgG phycoerythrin-labeled antibody, at 4°C in the dark for 30 minutes. For intracellular staining, cells were pre-fixed with 1% formaldehyde and permeabilized with IC Perm Buffer (Biosource) supplemented with 4% BSA. Staining was then performed as described above. FACS was carried out using FacScan and analyzed with the CellQuest software (Becton Dickinson, San Jose, CA, USA). Results were expressed as the mean fluorescence intensity ratio, defined as the mean fluorescence intensity of cells stained with an antigen-specific antibody divided by the mean fluorescence intensity of cells incubated with the isotype control antibody.

### Animal experiments

Male New Zealand White rabbits (body weight 2.7 to 3.0 kg) were obtained from the Elevage scientifique des Dombes (Romans, France). They were placed in individual cages and were fed *ad libitum *with water and nutriments, according to current recommendations mentioned in the *Guide for the Care and Use of Laboratory Animals *(National Institutes of Health No. 92-23, revised 1985). The Dijon Faculty of Medicine Ethical Committee approved the experimental protocol.

**Figure 1:**
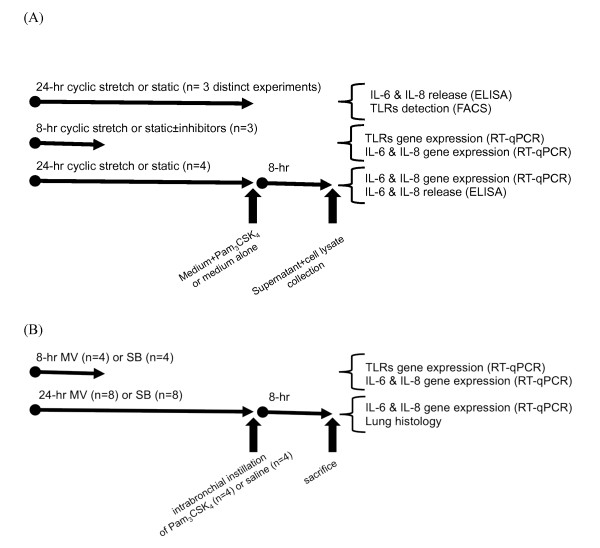
**Experimental design description**. **(a) ***In vitro *experiments. **(b) ***In vivo *experiments. FACS, fluorescence-activated cell sorting; MV, mechanical ventilation; qPCR, quantitative PCR; SB, spontaneously breathing; TLR, toll-like receptor.

In a first set of experiments, animals were randomly allocated to the SB group (*n *= 4) or the MV group (*n *= 4) (Figure 1). Animals from the former group were immediately sacrificed by a lethal dose of penthotal, whereas the latter were orally intubated as previously described [8,9]. The animal was then connected to a pressure-controlled ventilator (Babylog^®^; Drager, Lübeck, Germany). MV was performed in the supine position with continuous infusion of ketamine and pancuronium bromide. The peak inspiratory pressure was set at 12 cmH_2_O with zero end-expiratory pressure, a respiratory rate of 30/minute and an inspired fraction of oxygen of 0.5. The tidal volume was measured at the onset of MV by placing a pneumotachometer between the endotracheal tube and the ventilator circuit (12.6 ± 0.8 ml/kg). This corresponds to mild-stretch MV. Animals from the MV group were submitted to MV during an 8-hour period before being sacrificed. In both groups, animals were exsanguinated by venous puncture and lungs were harvested for RNA extraction. Additional samples were obtained for microscopic examination. A tissue sample of 1 cm^3 ^was fixed in formalin and embedded in paraffin. Four-micrometer sections were obtained and colored with hematoxylin and eosin.

In a second set of experiments, rabbits were orally intubated as described above and subjected to MV for a 24-hour period before being given intrabronchially either 500 μg Pam_3_CSK_4 _diluted in 2 ml isotonic saline or vehicle alone, through a silicon catheter introduced into the tracheal tube and pushed until one lower lobe bronchus was reached. The animals were then kept under MV during an 8-hour period before being sacrificed. An arterial catheter was inserted in most of these animals for blood sampling and blood pressure monitoring. Controls were SB animals that received 500 μg Pam_3_CSK_4 _and were allowed to go back to their cage before being sacrificed 8 hours later.

### Statistical analysis

Data are presented as the mean (standard deviation). The Mann-Whitney U test or the Kruskal-Wallis test was used to compare continuous variables whether the number of groups was two or more. *P *≤0.05 was considered significant.

## Results

### *In vitro *studies

#### Cyclic stretch induces the expression of Toll-like receptors in lung epithelial cells

**Figure 2:**
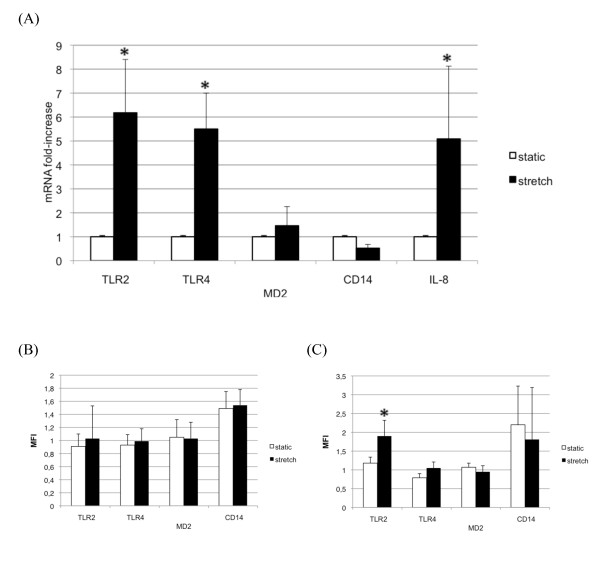
**Pathogen recognition receptor expression in cells submitted to cyclic stretch or kept in static condition**. **(a) **mRNA fold induction in A549 cells stretched for 8 hours or kept in static condition. All values are shown as the fold increase compared with control static cells normalized to 1. IL-8 mRNA levels induced by stretch served as a positive control. Both toll-like receptor (TLR)2 and TLR4 mRNA levels were markedly increased by cyclic stretch. MD-2 and CD14 mRNA levels were not significantly modified by stretch. Results are expressed as mean ± standard deviation of three distinct experiments. **(b) **Surface expression of TLR2, TLR4, MD-2 and CD14 by fluorescence-activated cell sorting (FACS) in A549 cells submitted to a 24-hour cyclic stretch or in cells kept in static condition. Except for CD14, none of these receptors showed significant surface expression. **(c) **Intracellular expression of TLR2, TLR4, MD-2 and CD14 by FACS in permeabilized A549 cells submitted to a 24-hour cyclic stretch or in cells kept in static condition. TLR2 was detected in permeabilized A549 cells after 24 hours of cyclic stretch. CD14 was detected, but not increased, by cyclic stretch. Neither TLR4 nor MD-2 proteins were detected by FACS in permeabilized cells, regardless of the experimental condition. Results are expressed as the mean fluorescence index (MFI) ± standard deviation of three distinct experiments. *P < 0.05 between static and stretched cells.

A sixfold increase in epithelial cell content in TLR2 mRNA was detected by quantitative PCR in stretched A549 cells when compared with cells kept in static condition (Figure 2a). TLR4 mRNA was increased to the same extent in stretched cells but MD-2 and CD14 mRNA levels remained unchanged.

#### Cyclic stretch increases intracellular TLR2 in lung epithelial cells

Cells were harvested after a 24-hour cyclic stretch for FACS analysis. No TLR2 was detected in A549 cells in a static condition in nonpermeabilized cells as well as in permeabilized cells. After 24 hours of cell stretching, TLR2 became detectable in permeabilized epithelial cells and to a lesser extent in nonpermeabilized cells (Figure 2b, c). TLR4 and MD-2 remained undetectable in intact or permeabilized cells regardless of the mechanical stress. Finally, cyclic stretch did not influence CD14 expression. The promonocytic human THP1 cells expressing TLR2, TLR4, CD14 and MD-2 were used as positive controls for the mAbs used in the experiments (data not shown).

#### Cyclic stretch increases the inflammatory response of lung epithelial cells to bacterial lipopeptide

**Figure 3:**
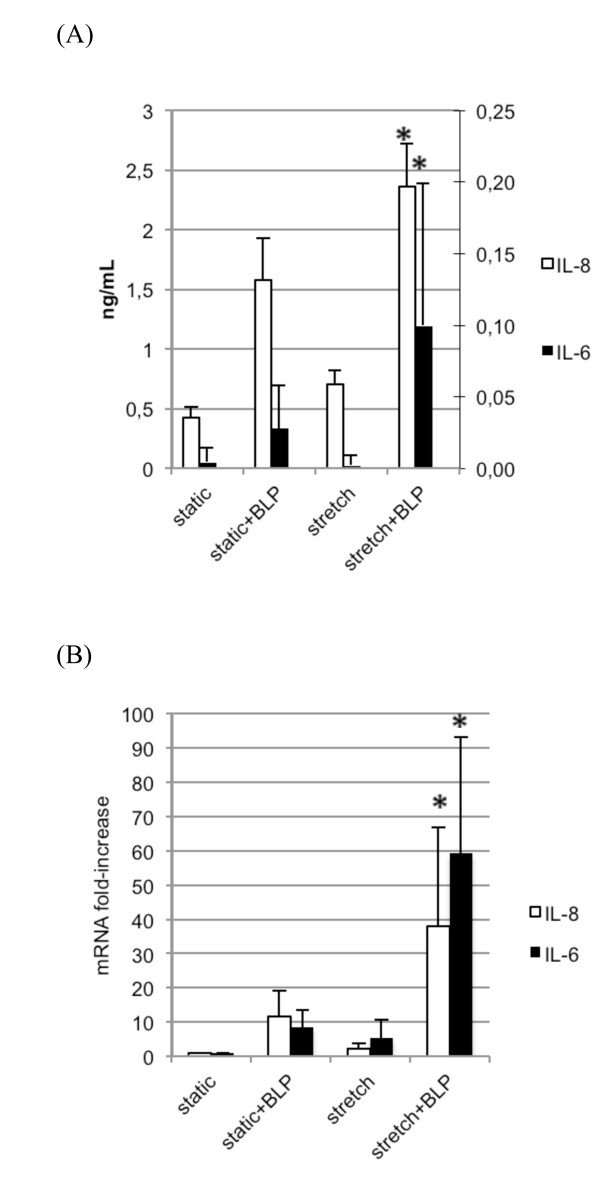
**Inflammatory mediator production by A549 cells stimulated with Pam_3_CSK_4 _in static and cyclic stretch conditions**. **(a) **IL-6 and IL-8 protein concentrations in conditioned supernatants from cells stretched for 24 hours or kept in static condition, and subsequently stimulated for 8 hours with bacterial lipopeptide (BLP). Cyclic stretch increased the inflammatory response of A549 cells in response to BLP. **(b) **IL-6 and IL-8 mRNA levels in human type II-like A549 alveolar cells stretched for 24 hours or kept in static condition, and subsequently stimulated for 8 hours with BLP. mRNA levels were normalized to the expression of GAPDH mRNA. All values are reported as the fold increase compared with control static cells normalized to 1. For both inflammatory markers, a clear synergistic effect between cyclic stretch and BLP is observed. Results are expressed as the mean ± standard deviation of four distinct experiments. *P < 0.05 between static and stretched cells stimulated with BLP.

A549 cells submitted to a 24-hour stretch released higher levels of IL-6 and IL-8 in response to an additional 8-hour stimulation by the synthetic BLP Pam_3_CSK_4 _than did cells not submitted to cyclic stretch (Figure 3a). A synergistic effect between cyclic stretch and Pam_3_CSK_4 _was also observed at the IL-6 and IL-8 mRNA level (Figure 3b).

#### Cyclic stretch-induced upregulation of TLR2 depends on the p38 MAPK signaling pathway

**Figure 4:**
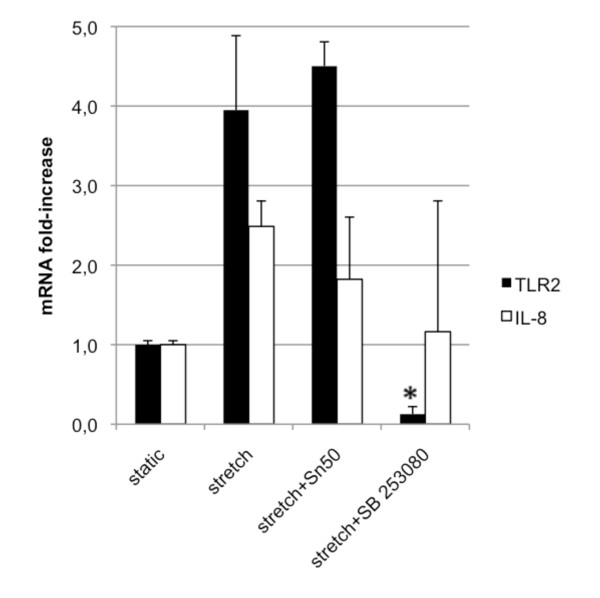
**Toll-like receptor 2 expression in A549 cells stretched with various inhibitors**. IL-8 mRNA levels were measured as a positive control of cell activation by cyclic stretch. mRNA levels were normalized with the expression of GAPDH mRNA. All values are reported as the fold increase compared with control static cells normalized to 1. SB203580 significantly reduced the toll-like receptor (TLR)2 mRNA level induced by stretch, whereas Sn50 had no effect. The opposite was observed for IL-8 expression induced by cyclic stretch. Results are expressed as the mean ± standard error of the mean of three distinct experiments. *P < 0.05 between stretched cells with or without SB 203580. SB, spontaneously breathing.

To address the question of the signaling pathway by which cyclic stretch increased TLR2 mRNA, we tested the effects of a NF-κB inhibitor (Sn50, 18 μM), and a p38 MAPK-specific inhibitor (SB203580, 1 μM) on cyclic stretch-induced *IL-8 *expression. The stretch-induced *TLR2 *expression in A549 cells was markedly reduced by the p38 MAPK inhibitor, whereas the NF-κB blocker did not inhibit stretch-induced *IL-8 *(Figure 4). Notably, we checked that SB203580 did not alter cell viability using a 3-[4,5-dimethylthiazol-2-yl]-2,5-diphenyl tetrazolium bromide assay (MTT) test (data not shown).

### *In vivo *studies

#### Mechanical ventilation in healthy rabbits causes a marked increase of lung TLR2 mRNA

**Figure 5:**
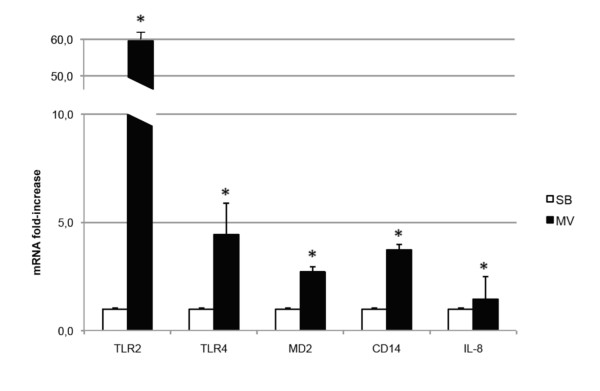
**Pathogen recognition receptor expression in rabbits submitted to mechanical ventilation or kept spontaneously breathing**. Lung IL-8 mRNA levels were measured as a positive control of lung inflammation induced by mechanical ventilation (MV). mRNA levels were normalized with the expression of GAPDH mRNA. All values are shown as the fold increase compared with spontaneously breathing (SB) rabbits normalized to 1. MV induced a strong increase of toll-like receptor (TLR)2 mRNA levels, higher than TLR4, MD-2 and CD14. Results are expressed as mean ± standard deviation. *P < 0.05 between SB and MV rabbits.

MV induced a marked increase in lung TLR2 mRNA, much greater than those observed for TLR4, MD-2, and CD14 (Figure 5).

**Figure 6:**
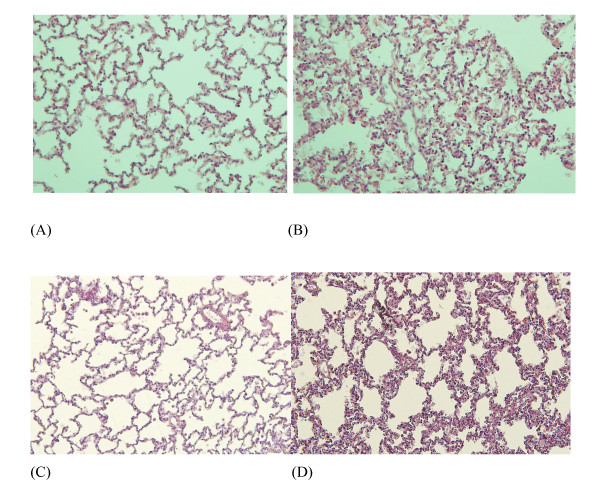
**Light photomicrographs of rabbit lungs in various conditions**. **(a) **Lung from a spontaneously breathing (SB) animal. **(b) **Rabbit lung after 32-hour mild-stretch mechanical ventilation (MV). **(c) **Lung from a SB animal following tracheal instillation of the synthetic bacterial lipopeptide (BLP), Pam_3_CSK_4_, and sacrificed 8 hours later. **(d) **Lung from an animal submitted to 24-hour mild-stretch MV followed by tracheal instillation of BLP and sacrifice 8 hours later. The main abnormalities were septal thickening and inflammatory cell infiltration in rabbits submitted to MV, increased by the presence of BLP. Representative fields, original magnification, x10; hematoxylin and eosin stain.

**Figure 7:**
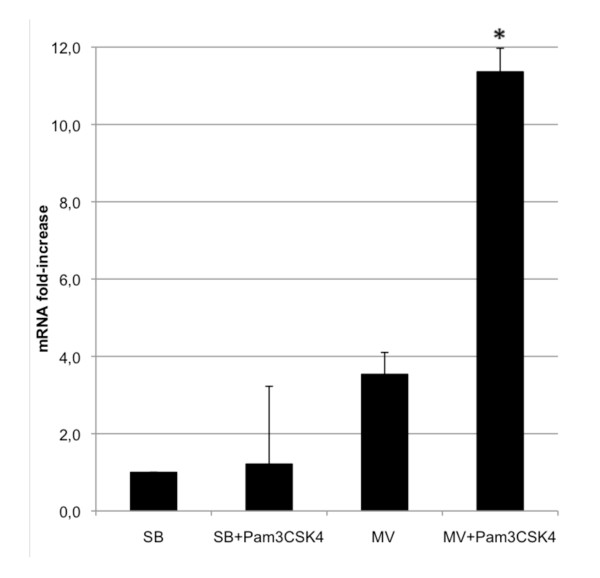
**IL-8 expression following instillation of Pam_3_CSK_4 _in rabbits submitted previously, or not, to mechanical ventilation**. IL-8 mRNA levels were normalized with the expression of GAPDH mRNA. Values are reported as the fold increase compared with control spontaneously breathing (SB) animals normalized to 1 (*n *= 4 per group). Results are expressed as the mean ± standard deviation. Lung response to bacterial lipopeptide (BLP) was markedly enhanced by previous mechanical ventilation (MV). *P < 0.05 between SB and MV stimulated rabbits.

In these ventilated rabbits, no macroscopic lung abnormalities were noted. Microscopic studies, however, showed features of mild lung injury, including septal thickening and inflammatory cell infiltration together with a small increase in IL-8 mRNA (Figures 6 and 7) - features absent in lungs from SB animals.

#### Mechanical ventilation increases lung inflammation induced by bacterial lipopeptide

Despite MV and lung insult by Pam_3_CSK_4_, hemodynamic stability and adequate blood oxygenation were achieved in our model (Table 1).

**Table 1:** Physiological parameters in rabbits after mild-stretch mechanical ventilation and lung insult

Experimental time	MAP (mmHg)	HR (/minute)	pH	PaO_2 _(mmHg)	PaCO_2 _(mmHg)
T1	89 (12.7)	202 (30.4)	7.40 (0.07)	213 (44)	22 (14)
T2	87 (13.1)	276 (29.7)	7.43 (0.10)	210 (42)	21 (10)

Physiological parameters recorded in rabbits after a 24-hour mild-stretch mechanical ventilation (T1) and 8 hours following the tracheal instillation of bacterial lipopeptide (T2). HR, heart rate; MAP, mean arterial pressure.

The endobronchial instillation of the BLP Pam_3_CSK_4 _induced more lung injury in rabbits previously ventilated for 24 hours compared with those who were SB during that period of time (Figure 6). These microscopic abnormalities included more inflammatory cell infiltration and more pronounced alveolar septa thickening.

Although Pam_3_CSK_4 _alone did not induce much IL-8 mRNA, we observed a clear synergistic effect between MV and BLP on lung IL-8 gene expression (Figure 7).

## Discussion

Herein, we show that cyclic stretch of lung cells and MV in rabbits upregulate *TLR2*. MV-dependent *TLR2 *upregulation was associated with a marked increase in lung cell activation and lung injury after a challenge with Pam_3_CSK_4_, a classical TLR2 agonist. These results are consistent with cyclic stretch increasing lung cell reactivity to TLR2 bacterial agonists. Prestretching type II-like human alveolar A549 cells induced a marked increase in the induction of proinflammatory mediators such as IL-6 and IL-8 in response to BLP; a synergistic effect was observed between MV and TLR2 activation by Pam_3_CSK_4_. We cannot, however, exclude that the increased expression of *TLR2 *by A549 cells is solely and directly caused by cyclic stretch. Actually, the release of inflammatory mediators by stretched cells might account at least in part for the observation reported herein [19].

We failed to detect TLR2 at the surface of epithelial cells by FACS. Depending on cell types, TLRs can be expressed at the surface or intracellularly. TLR4, for example, is expressed at the surface of myeloid cells but in intracellular compartments in epithelial cells such as A549, BEAS-2B cells, intestinal cells as well as endothelial cells [20,21]. Similarly, TLR2 - which is also expressed at the plasma membrane in myeloid cells - is found intracellularly in corneal epithelial cells [22]. Using a permeabilization protocol, we showed that cyclic stretch induced intracellular TLR2 upregulation after cyclic stretch in A549 cells. TLR2 mRNA was also increased by cyclic stretch in A549 cells.

Cyclic stretch has been shown to induce proinflammatory gene transcription in lung epithelial cells via either NF-κB or MAPK pathways [7]. We therefore tested the effect of pharmacological inhibitors of the NF-κB and the p38 MAPK signalization pathways on cyclic stretch-dependent induction of *TLR2*. The p38 MAPK inhibitor (SB203580) abrogated cyclic stretch-induced TLR2 gene expression, whereas NF-κB had no effect. These findings are consistent with the fact that the MAPK pathway is critical in the cyclic stretch-induced cell activation. These data also indicate that *TLR2 *regulation is not entirely dependent on the NF-κB pathway, depending on the stimulus [23]. The involvement of the p38 MAPK pathway in *TLR2 *regulation by cyclic stretch is suggestive of TLR2 mRNA stabilization (post-transcriptional effect) rather than *de novo TLR2 *transcription, but this remains to be ascertained using TLR2 transcription assays.

We next tested whether these *in vitro *findings could be reproduced in an animal model. We showed that mild-stretch MV induced a marked upregulation of lung *TLR2*. Furthermore, the treatment with BLP of rabbits ventilated for 24 hours induced a marked IL-8 expression into the airway and significant lung injury, as compared with SB rabbits.

This is the first report showing the MV-induced upregulation of *TLR2*, and a synergistic effect between MV and BLP. These results are reminiscent of those found with the LPS/CD14/TLR4 pathway [3-6,24], and highlight the increased reactivity of lung cells to Gram-positive bacterial products and bacteria induced by MV via the TLR2 pathway [25,26]. Moreover, in contrast with previous findings, we observed that *TLR2 *and *TLR4 *could be differentially upregulated by cyclic stretch. Accordingly, the *TLR2 *upregulation was strikingly more pronounced, especially in the *in vivo *model. Interestingly, similar findings were obtained *in vitro *in human endothelial cells submitted to either a laminar or a turbulent flow [17]. Such data suggest that differences exist between TLRs with respect to their expression regulation by abnormal mechanical strains.

Our work adds to a growing body of literature implicating pattern-recognition receptors of the innate immunity in the development of ventilator-induced lung injury. A large tidal volume MV increased *TLR4 *and *TLR2 *expression in the lungs of preterm lambs, but the effect of the corresponding agonist was not tested [27]. Others have demonstrated that lung hyperinflation increased *TLR4 *expression in rats with abdominal sepsis [25]. In contrast, *TLR4 *knockout mice were protected against ventilator-induced lung injury due to lung hyperinflation [28]. Interestingly, it was shown that even protective MV applied to healthy mouse lungs could modify TLR expression [29].

Any clinical translation should be made cautiously, however, since the chosen ventilator settings were not in accordance with the current guidelines. The aim was to cause overdistension within healthy lungs, a goal difficult to achieve with the recommended settings (that is, 6 to 8 ml/kg and positive end-expiratory pressure). Such lung-protective settings, however, could lead to regional overdistension when applied to injured lungs [30,31]. Another limitation is that we have not studied the relationship between lung stretch magnitude and *TLR2 *expression since only one degree of cell elongation as well as one tidal volume value were tested. We cannot therefore conclude definitively that *TLR2 *expression is stretch dependent.

## Conclusions

Cyclic stretch mimicking MV *in vitro *induced *TLR2 *expression and enhanced TLR2 reactivity to BLP of epithelial human cells, most probably through a p38 MAPK-dependent effect. These findings were in part reproduced in a rabbit model, where mild-stretch MV increased lung injury in response to the instillation of a TLR2-dependent BLP. Translated into the clinical arena, our findings suggest that the increase of the *TLR2 *expression of the lung epithelia caused by MV may prime the lung, which in turn will overreact to the presence of colonizing or infecting Gram-positive bacteria within the airway and worsen lung injury. The potential impact on lung bacterial clearance warrants further studies.

## Key messages

• Mechanical ventilation magnifies lung inflammatory response to a Gram-positive bacterial product, and in turn worsens lung injury.

• The stretch-dependent *TLR2 *upregulation could account for such findings.

• The p38 MAPK pathway could play a critical role.

## Abbreviations

BLP: bacterial lipopeptide; BSA: bovine serum albumin; DMEM: Dulbecco's modified Eagle's medium; ELISA: enzyme-linked immunosorbent assay; FACS: fluorescence-activated cell sorting; FCS: fetal calf serum; FIO_2_: fraction of inspired O_2_; IL: interleukin; LPS: lipopolysaccharide; mAb: monoclonal antibody; MAPK: mitogen-activated protein kinase; MV: mechanical ventilation; NF: nuclear factor; PBS: phosphate-buffered saline; PCR: polymerase chain reaction; RT: reverse transcriptase; SB: spontaneously breathing; TLR: toll-like receptor.

## Competing interests

The authors declare that they have no competing interests.

## Authors' contributions

PEC, PT, SDB and JP designed the experiments, carried out the study, analyzed the results and drafted the manuscript. DC, IDS, JD and PC participated in the study. All of the authors read and approved the final version of the manuscript.
